# A Two-Way Proteome Microarray Strategy to Identify Novel *Mycobacterium tuberculosis*-Human Interactors

**DOI:** 10.3389/fcimb.2019.00065

**Published:** 2019-03-28

**Authors:** Tingming Cao, Lingna Lyu, Hongyan Jia, Jinghui Wang, Fengjiao Du, Liping Pan, Zihui Li, Aiying Xing, Jing Xiao, Yu Ma, Zongde Zhang

**Affiliations:** Beijing Key Laboratory for Drug Resistant Tuberculosis Research, Beijing Tuberculosis and Thoracic Tumor Research Institute, Beijing Chest Hospital, Capital Medical University, Beijing, China

**Keywords:** human proteome microarray, *Mtb* proteome microarray, host-pathogen interaction, NRF1, SMAD2

## Abstract

Tuberculosis (TB) is still a serious threat to human health which is caused by mycobacterium tuberculosis (*Mtb*). The main reason for failure to eliminate TB is lack of clearly understanding the molecular mechanism of *Mtb* pathogenesis. Determining human *Mtb*-interacting proteins enables us to characterize the mechanism and identify potential molecular targets for TB diagnosis and treatment. However, experimentally systematic *Mtb* interactors are not readily available. In this study, we performed an unbiased, comprehensive two-way proteome microarray based approach to systematically screen global human *Mtb* interactors and determine the binding partners of *Mtb* effectors. Our results, for the first time, screened 84 potential human *Mtb* interactors. Bioinformatic analysis further highlighted these protein candidates might engage in a wide range of cellular functions such as activation of DNA endogenous promoters, transcription of DNA/RNA and necrosis, as well as immune-related signaling pathways. Then, using *Mtb* proteome microarray followed His tagged pull-down assay and Co-IP, we identified one interacting partner (Rv0577) for the protein candidate NRF1 and three binding partners (Rv0577, Rv2117, Rv2423) for SMAD2, respectively. This study gives new insights into the profile of global *Mtb* interactors potentially involved in *Mtb* pathogenesis and demonstrates a powerful strategy in the discovery of *Mtb* effectors.

## Introduction

Tuberculosis (TB) is a chronic infectious disease caused by the intracellular bacterial pathogen *Mycobacterium tuberculosis* (*Mtb*). According to a WHO report, there were an estimated 10.4 million new cases of TB and 1.67 million TB-related deaths in 2016 (World Health Organization (WHO), 2017). Moreover, emergence of multi-drug resistant (MDR) and extensively-drug resistant (XDR) strains of *Mycobacterium*, as well as co-infection with retroviruses like HIV has further complicated TB treatment (Goldberg et al., 2012; Chang and Yew, 2013). To overcome this menace, comprehensive understanding of the molecular crosstalk between the invading pathogen and its human host will be extremely important to identify targets for the effective treatment of TB.

*Mtb* is one of the most successful pathogens that can survive and replicate within the macrophages of infected people (Cambier et al., 2014). After invasion of *Mtb*, macrophages can generate an immune response that utilizes effectors such as anti-bacterial peptides, hydrolases, and toxic reactive oxygen and nitrogen intermediates (Alipoor et al., 2016). In addition to macrophages, T-cells have been also shown to be involved in host cell immune response against *Mtb* (Gonzalez-Juarrero and Orme, 2001). However, *Mycobacterium* has developed multiple strategies to evade elimination by the host immune response and causes disease, such as arrest of phagosome-lysosome fusion, inhibition of apoptotic/autophagic pathways of invading macrophages, and promotion of neutralization of reactive nitrogen and oxygen intermediates (Russell, 2001; Gutierrez et al., 2004; Flannagan et al., 2009).

The protein-protein interactions (PPI) between *Mtb* and the host cell are often involved in the bacteria's pathogenesis and persistence in the host (Konig et al., 2008; Dyer et al., 2010). However, such systematic inter-species PPI are not readily available. Experimental studies have uncovered and characterized a few PPI between *Mtb* and humans (Table S1) (Mueller-Ortiz et al., 2001; Vergne et al., 2005; Deghmane et al., 2007; Bach et al., 2008; Danelishvili et al., 2010; Sun et al., 2010; Wong et al., 2011; Byun et al., 2012; Kim et al., 2012; Mehra et al., 2013; Sreejit et al., 2014; Wang et al., 2015, 2016; Dziadek et al., 2016). There are two main approaches applied to discover these interactions, including yeast two-hybrid (Y2H) system and affinity pull-down (AP) coupled with mass spectrometry (MS) (Jäger et al., 2011; Mehra et al., 2013; Rajagopala et al., 2014; Dziadek et al., 2016). However, the sensitivity of Y2H is estimated at only ~20%, and AP/MS can detect proteins in complexes but is unable to distinguish direct from indirect interactions when several proteins are purified together. Furthermore, these methods are time-consuming and expensive, especially when adopted in high-throughput mode (Liu et al., 2012). Recently, several computational approaches have been developed to predict host-pathogen PPI based on the sequence method of homology, interologs, interacting domain/motif, and structure (Rapanoel et al., 2013; Zhou et al., 2013). However, most of these works lack stringent verification. Thus, the accuracy of computational approaches in predicting host-pathogen PPI is largely unknown.

In this study, we attempt to fill this gap by identifying the global human interactors with *Mtb* using an unbiased, comprehensive protein-chip based approach and demonstrating a powerful strategy in the discovery of *Mb* effectors. Our results could provide potential targets for a further study of pathogenesis of *Mtb* and drug development for TB.

## Experimental Procedures

### *Mtb* Secreted Protein (SP) and Cellular Protein (CP) Sample Preparation

*Mtb* SP and CP were extracted as described by Andersen and his colleagues with minor modifications (Andersen et al., 1991). In short, *Mtb* H37Rv were cultured in Middlebrook 7H9 broth (BD-Defico, USA) added with 10% OADC and 0.05% Tween 80 until the logarithmic phase (usually 3 weeks). The bacteria were collected and heat inactivated at 90°C for 30 min. The cellular proteins were extracted by using multigelation for 3 times and ultrasonication under conditions of 800 W, 2 s/2 s for 40 min followed by centrifugation and collection of supernatant. The cultures were centrifuged and the supernatants were sterile filtered. After concentration using Amicon ultra-15 (3 kDa) (Millipore, USA), the supernatants were extensively washed in PBS while still in ultrafiltration cell. The protein content was determined by coomassie brilliant blue staining. Then SP and CP were diluted to 0.3 mg/ml and labeled with CyDye Protein Labeling Cy3/Cy5 (GE-Healthcare, USA) respectively. Two microliters of the proteins were loaded on Nitrocellulose(NC) membrane followed by BSA blocking and PBS washing, then the signal was detected by a fluorescent scanner to evaluate the laveling efficiency.

### Human Proteome Microarray Fabrication

HuProt™ version 3.0 microarray were purchased from CDI Laboratories Inc (USA). It contains more than 19,000 unique proteins which encompasses 15,581 unique human genes and 124 unique mouse gene symbols. Recombinant proteins are expressed in the yeast *S. cerevisiae*, purified, and printed on glass slides in duplicate, Control proteins, including histone H1, histone H2, histone H3, histone H4, BSA, and biotinylated BSA, GST, IgM, IgG were also spotted in duplicate. The printed microarrays were stored at −80°C prior to use.

### Identification of *Mtb* SP Interactors (SPI) and CP Interactors (CPI) Using the Human Proteome Microarrays

Proteome microarrays were blocked with blocking buffer (5% BSA in 0.1% Tween 20 TBST) for 1.5 h at room temperature with gentle agitation. SP and CP samples were diluted to 1 μg/ml and incubated on the proteome microarray at room temperature for 2 h. The microarrays were each washed with TBST three times for 5 min, and followed by three quick washes in ddH_2_O. The microarrays were spun dry at 250 g for 3 min and were scanned with a GenePix 4200A microarray scanner (Molecular Devices, CA, USA) to visualize and record the results. The proteome microarray data were extracted with GenePix Pro 6.0 (Molecular Devices, CA, USA) and processed as previously described (Wang et al., 2012).

### Bioinformatic Analysis of Human *Mtb* Interactors

The protein candidates were classified using the PANTHER classification system with default settings (Mi et al., 2007). To identify the enrichment of the candidates, the potential candidates were analyzed using DAVID 6.8 (Huang et al., 2009a,b). Significantly enriched categories in the subontology of the KEGG pathway with a *P* value < 0.05 were chosen. We also performed analysis on functions, pathways and networks using the Ingenuity Pathway Analysis (IPA) software (https://www.qiagenbioinformatics.com), which were evaluated by *p*-value (*p*-value < 0.05). The protein symbol of each candidate was used as input data.

### Identification the *Mtb* Effectors Using *Mtb* Proteome Microarray

To identify the *Mtb* targets of these human interactors, representative proteins SMAD2 and NRF1 were cloned into the pCMV-C-myc vector, respectively, and purified as previously reported (Li et al., 2013). The protein concentration was determined by standard silver staining. Then the myc-tagged SMAD2 and NRF1 were labeled with Cy3 and diluted to 4 μg/ml. *Mtb* proteome microarrays (BC Biotech, China) were blocked for 3 h at 4°C with shaking in blocking buffer (3% BSA in PBS). The protein samples were incubated with microarrays overnight at 4°C with shaking, then washed three times in PBS (pH 7.4) with 0.1% Tween 20 (PBST) followed by twice ddH2O quick washes. Microarrays were spun dry at 250 g for 3 min and then scanned with a GenePix 4200A microarray scanner. Data were analyzed with GenePix Pro 6.0 and processed as previously described (Jeong et al., 2012).

### His Tagged Pull-Down Assay and Co-Immunoprecipitation

To validate the interactions between screened *Mtb* effectors and human SMAD2 or NRF1, His tagged *Mtb* proteins were purified as previously described (Biertümpfel et al., 2010). PMA-differentiated THP-1 cells lysed in HEPEs lysis buffer (50 mM HEPEs pH 7.5, 150 mM NaCl, 1 mM EDTA, 1 mM EGTA, 10% Glycerol, 1% TritonX-100, 10 μM ZnCl_2_) supplemented with 0.5 mM PMSF (Sigma-Aldrich, Saint Louis, USA). The His pull-down were performed as previously described (Zhang et al., 2009). Protein separation and detection were performed using an automated capillary electrophoresis system (Simple Western system and Compass software; proteinsimple; USA). Antibodies against the following proteins were used; anti-NRF1 (#46743, CST, 1:50), anti-SMAD2 (#5339, CST, 1:500) and anti-His-tag (#12698, CST, 1:100). Signals were detected by HRP-conjugated secondary anti-rabbit antibody and were visualized using Compass software. For Co-IP, Flag-Rv2117, -Rv2423, and -Rv0577 were overexpressed in HEK293T cells and the whole-cell lysates were immunoprecipitated with anti-Flag M2 agarose (Sigma, St Louis, MO, USA) as described previously(Guo et al., 2003). Samples were separated by SDS-PAGE and detected by immunoblotting with antibodies anti-Flag M2 (#14793, CST, 1:1000), anti-SMAD2 (1:2000) and anti-NRF1 (1:2000).

### Experimental Design and Statistical Rationale

The experiment was designed to conduct two separated proteome microarray assays. The proteome microarray data were extracted with GenePix Pro 6.0 and processed as previously reported (Jeong et al., 2012; Wang et al., 2012). For human proteome microarray data analysis, the signal-to-noise ratio (SNR) was defined as the ratio of median of foreground signal (F-median) deducted of median of Background signal (B-median) to B-median [(F-median-B-median)/B-median] and was calculated for each protein. The SNR of each protein was averaged for the two duplicated spots. To call the final potential candidates, the cut-off value was set as SNR ≥ 2. For *Mtb* proteome microarray data analysis, the signal to noise ratio (SNR) was defined as F-median/B-median. Z-score of the proteins was calculated to normalize signal intensity for any pair of spots. To call the positive binding partners, the cutoff was set as SNR ≥ 1.4 fold above the background and Z-score ≥ 3.0.

## Results

### Screening of Human Proteins Interacted With *Mtb* Proteins Using Human Proteome Microarray

**Figure 1:**
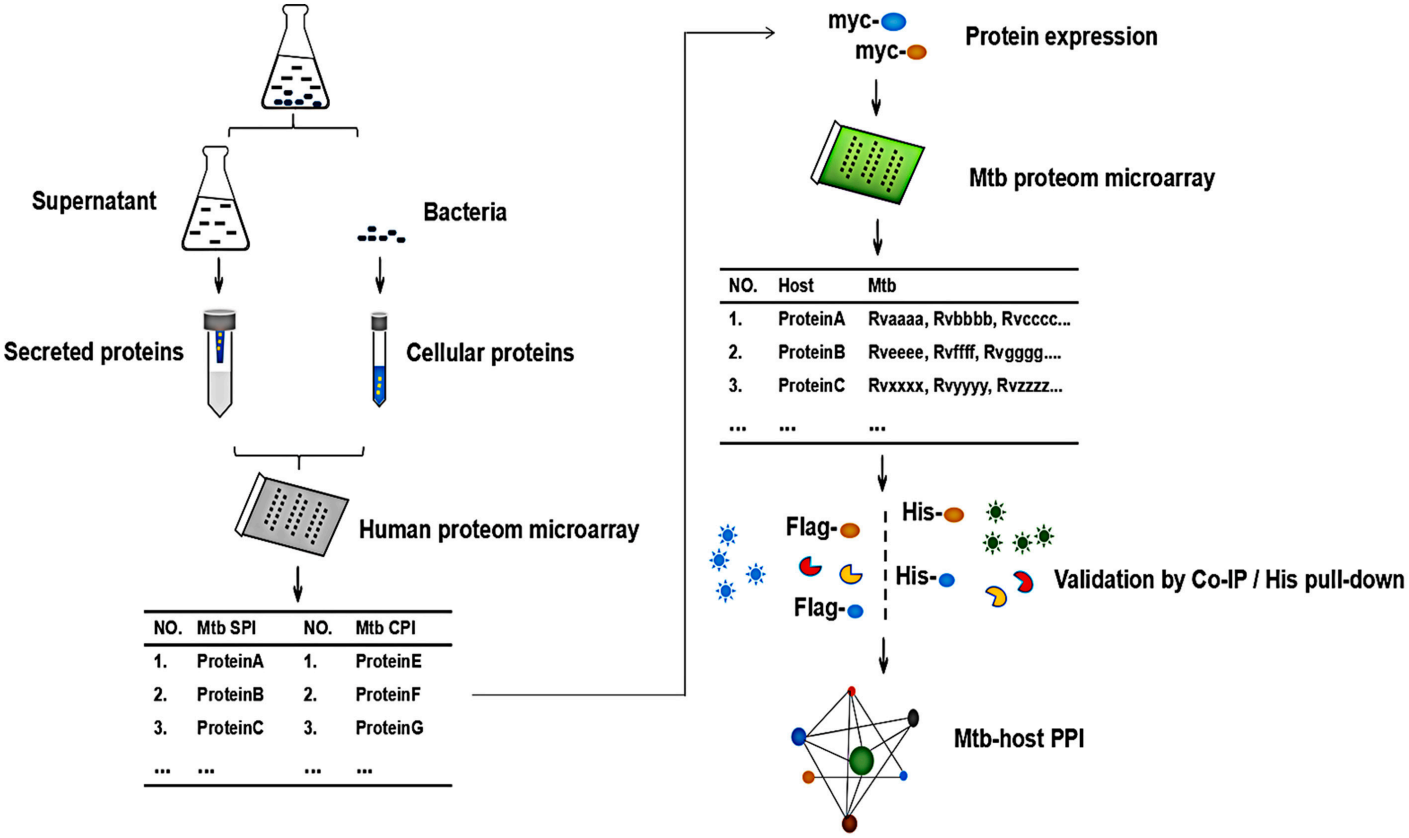
The schematic diagram of the proteome microarray strategy for identification of *Mtb*-human PPI. Briefly, the *Mtb* secreted protein (SP) and cellular protein (CP) were prepared and labeled with distinct fluorescein, respectively. After incubated with human proteome microarray, the potential *Mtb* interactors were screened. To call for *Mtb* effectors, human proteins were expressed and purified *in vitro*, reversely incubated with *Mtb* proteome microarray. Following experimental validation by His pull-down assay and Co-IP determined the interactions and the *Mtb*-host PPI.

In order to globally identify human proteins that directly interacted with *Mtb* proteins, we designed the experiment as shown in Figure 1. Briefly, The *Mtb* SP and CP were prepared and labeled with distinct fluorescein, respectively. After being incubated with human proteome microarray, the potential *Mtb* interactors were screened. To call for *Mtb* effectors, human proteins were expressed and purified *in vitro*, reversely incubated with *Mtb* proteome microarray to find out its binding partners in *Mtb*. Further validation of His pull-down assay and Co-IP were performed to verify these interactions between *Mtb* effectors and its human interactors so as to identify the *Mtb*-host PPI.

**Figure 2:**
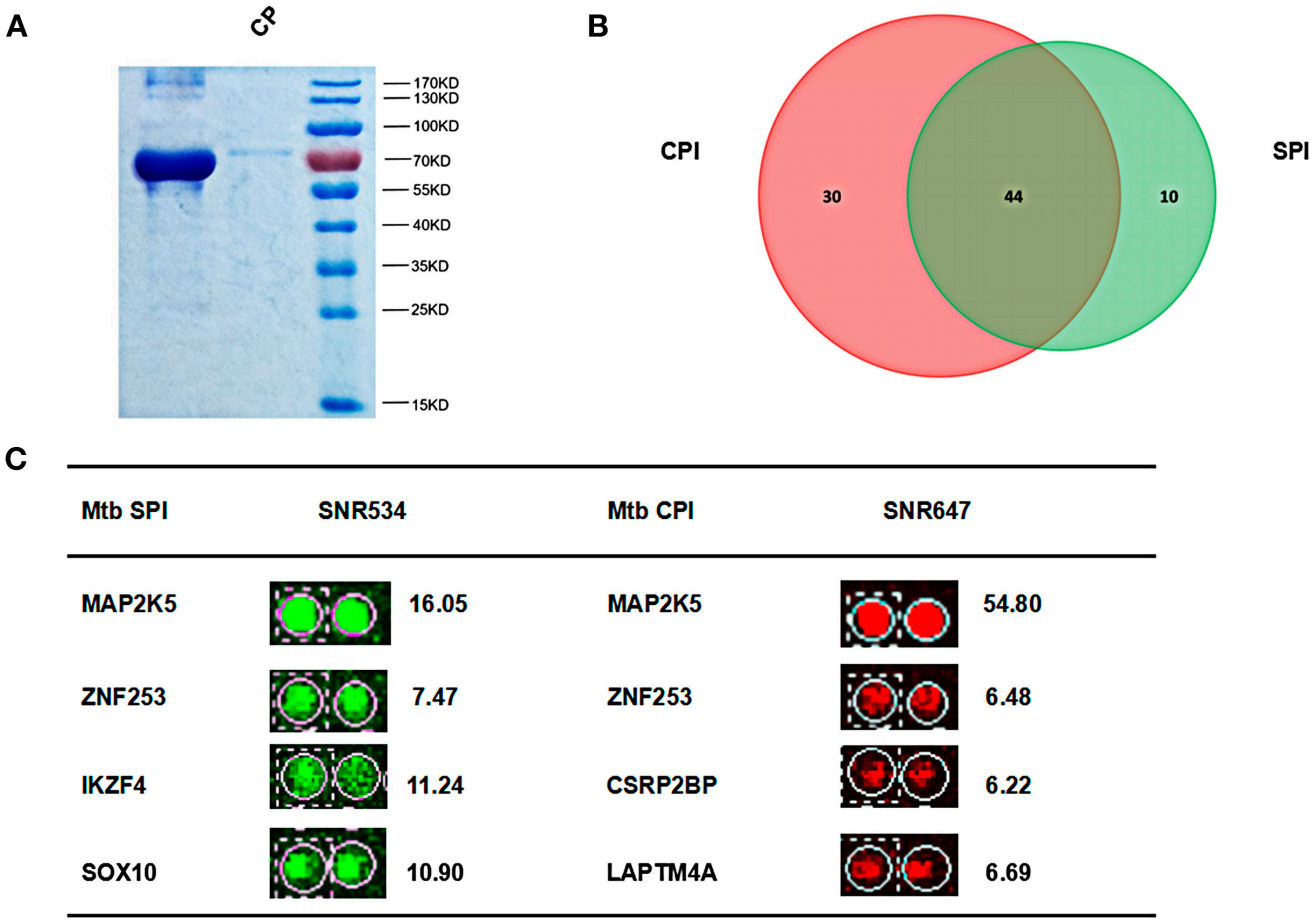
Identification of *Mtb* SPI and CPI on a human proteome microarray. **(A)** Extraction and purification of *Mtb* SP and CP. The proteins were detected by SDS-PAGE followed by coomassie brilliant blue staining. **(B)** The Venn diagram of *Mtb* SPI (green) and CPI (red). **(C)** Representatives of the newly identified *Mtb* SPI and CPI. The cutoff for calling positive candidates was set as signal-to-noise (SNR) ratio > 2.0. Experiments were performed in two replicates.

*Mtb* membrane proteins and secreted proteins are important effectors that may play important roles in host-pathogen interactions (Poirier and Av-Gay, 2012). To harvest all the *Mtb* proteins, we isolated *Mtb* SP from the supernatant of bacterial cultures and CP from the cell pellet, respectively (Figure 2A). After labeling with Cy3 or Cy5, the SP and CP were incubated with a human proteome microarray with 15,581 individually affinity-purified N-terminal GST-tagged human proteins. The signals at 532 nm and 647 nm were collected, and the reproducibility of the duplicate spots was assessed. To screen the potential human *Mtb* interactors, the cut-off was set as the signal-to-noise ratio (SNR) > 2. In this way, we identified 54 *Mtb* secreted protein interactors (SPI) and 74 human *Mtb* cellular protein interactors (CPI) with 44 proteins in common between the two parts and 84 proteins in total as potential *Mtb* interactors (Figure 2B). Four representatives of the potential interactors are shown in Figure 2C. Notably, there were 10 SP-unique interactors and 30 CP-unique interactors (Supplementary Figure 1), which suggested different regulatory roles for *Mtb* SP and CP during *Mtb* invasion of host cells.

### Categorization and Go Analysis of the *Mtb* SPI and CPI

**Figure 3:**
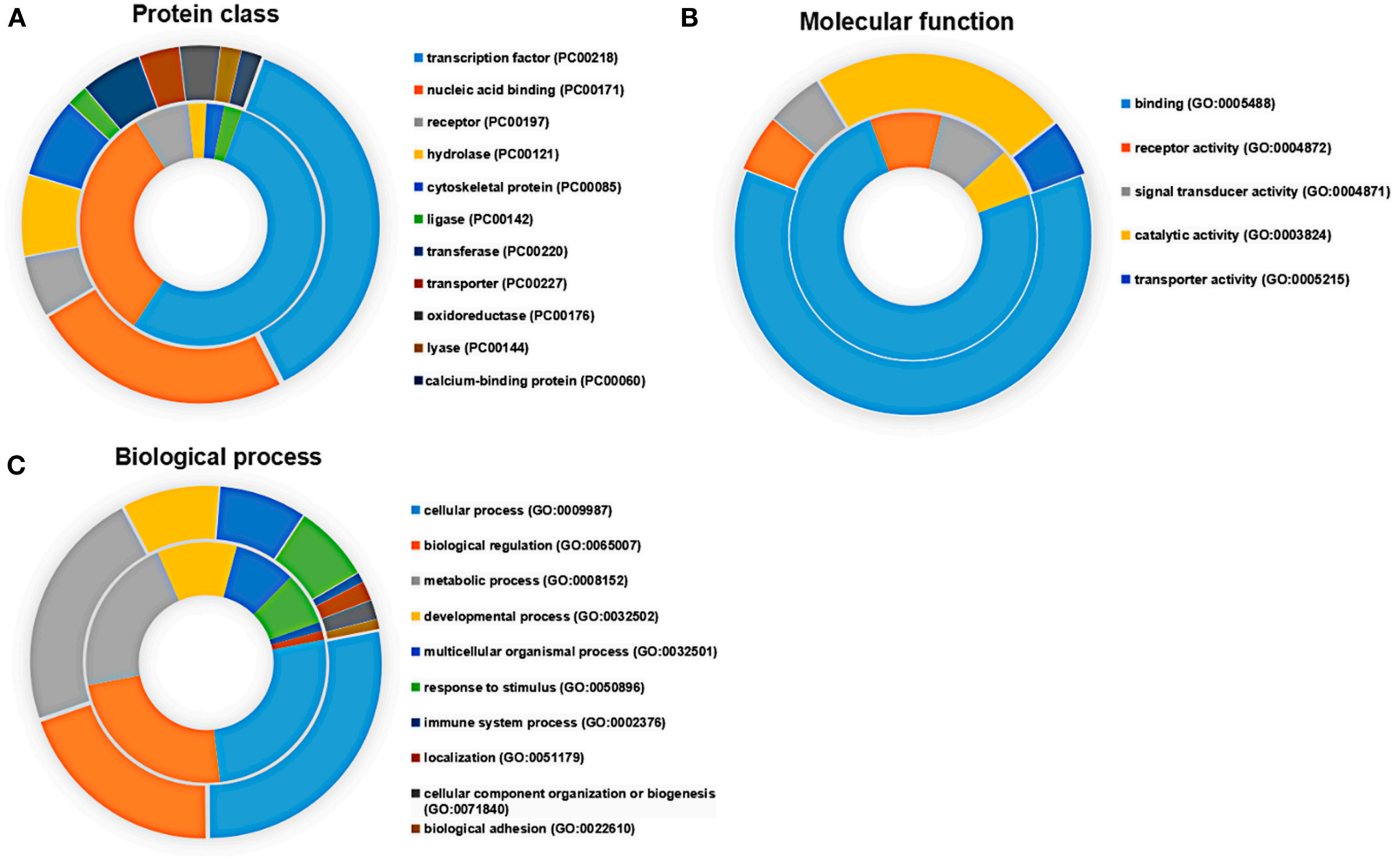
Functional distribution of the SPI (inner circle) and CPI (outer circle) Protein candidates were classified according to their **(A)** protein class, **(B)** molecular function, and **(C)** biological process using the PANTHER classification system (Mi et al., 2007) with default settings. Gene symbols were used as input.

Applying the online PANTHER classification system to the newly defined *Mtb* potential interactors, we determined the classification for protein class, specific biological process (BP) and molecular function (MF). The CPI could be classified into 11 protein classes, covering 6 items categorized for SPI (Figure 3A). The top two protein classes were transcription factor (PC00218) and nucleic acid binding (PC00171) for both SPI and CPI. Besides, the SPI could also be classified into 8 groups of BP, while additional 2 groups including cellular component organization or biogenesis (GO:0071840) and biological adhesion (GO:0022610) were defined in BP categories of CPI. The top three classes are cellular process (GO:0009987), biological regulation (GO:0065007), and metabolic process (GO:0008152), counting for 26.2, 23.8, and 21.4% in SPI and 27.9, 19.8, and 22.5% in CPI, respectively. As expected, response to stimulus (GO:0050896) and immune system process (GO:0002376) were defined in BP classes of both SPI and CPI (Figure 3C). Finally, the candidates of SPI could be defined as 4 classes of MF, which were also found in CPI, however, CPI had one more category marked as transporter activity (GO:0005215). Except for the top one MF class that was binding (GO:0005488), more candidates of CPI (23.1%) play roles in catalytic activity (GO:0003824) compared with those of SPI (6.3%) (Figure 3B). Next, we carried out gene ontology analysis by using DAVID as mentioned in EXPERIMENTAL PROCEDURES. Interestingly, we found that *Mtb* SP- and CP-unique interactors specifically preferred to bind with host transcription regulators, but they enriched into reverse GO BP terms (Table 1). Among the SP-unique interactors, PPARδ, BARX2, IRF2, MBD2, FOXD3 were enriched for term of negative regulation of transcription from RNA polymerase II (GO:0000122). While *Mtb* CP-unique interactors were significantly enriched for term of positive regulation of transcription (GO:GO:0045893). These results suggested that *Mtb* SP could arise most biological responses in host as part of the whole bacterial components. The different regulation of SP- and CP-unique interactors in host transcription might give some clues on the sequential and spatial regulatory role of *Mtb* after infection, battle with host immune system and finally persistence or death.

**Table 1:** The GO analysis of *Mtb* SP- and CP- unique interactors.

	**GO term**	**Genes**	***p*-value**
SP-unique interactors	GO:0000122: negative regulation of transcription from RNA polymerase II	PPARD,BARX2,IRF2, MBD2, FOXD3	3.55E-04
	GO:0000790: nuclear chromatin	PPARD, MBD2, FOXD3	3.82E-03
CP-unique interactors	GO:0006090: pyruvate metabolic process	LDHA, GLO1, PCK2	4.83E-04
	GO:0045893: positive regulation of transcription	CDX2,HNF4A,MAPK3, SOX4, RORA	6.63E-03
	GO:0071356: cellular response to tumor necrosis factor	RORA, NFE2L2, PCK2	1.16E-02
	GO:0043565: sequence-specific DNA binding	CDX2,HNF4A,RORA, NFE2L2	2.87E-02
	GO:0003700: transcription factor activity, sequence-specific DNA binding	CDX2,HNF4A,SOX4, RORA,NFE2L2	3.36E-02

### Function, Pathway and Network Analysis of the Global *Mtb* Potential Interactors

**Figure 4:**
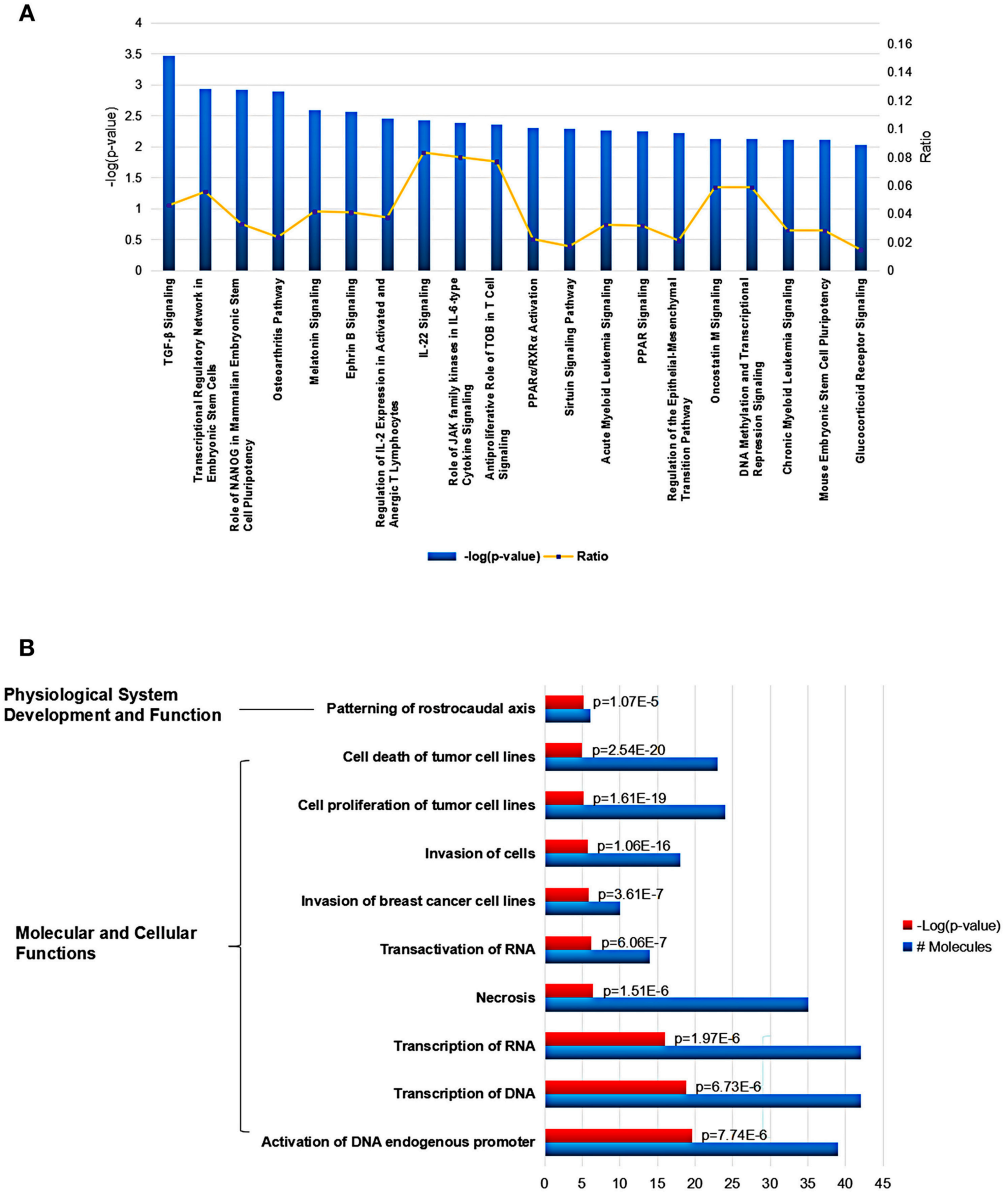
Cellular function and pathway analysis of the global *Mtb* interactors using IPA software. **(A)** The IPA canonical pathway analysis showed 20 significantly changed pathways (*p*-value < 0.05). **(B)** The top-10 significantly changed items covered the two IPA categories including the “Molecular and Cellular Functions,” and “Physiological System Development and Function”.

Using Ingenuity Pathway Analysis (IPA) software, we further gained an insight into the role for the global *Mtb* potential interactors in canonical pathways, cellular functions and networks. In canonical pathway analysis, these proteins were involved in the most significant item of “TGF-β signaling” which was responsible for early inhibition to facilitate *Mtb* clearance (Feruglio et al., 2017). Besides, other key immune-related signaling pathways were also defined such as “regulation of IL-2 expression in activated and anergic T Lymphocytes,” “IL-22 signaling,” “role of JAK family kinases in IL-6-type cytosine signaling,” and “antiproliferative role of TOB in T cell signaling.” The top 20 canonical pathway items could be found in Figure 4A and Table S2. Among all the IPA “Diseases and Bio Functions” items, the top 10 significantly changed items were showed and listed in Figure 4B and Table S3, which covered the two IPA categories including the “Molecular and Cellular Functions” and “Physiological System Development and Function.”

In addition, various networks were found to engage for the human *Mtb* interactors, the top three networks involved “Gene Expression, Embryonic Development, Organismal Development,” “Cellular Assembly and Organization, Endocrine System Disorders, Gastrointestinal Disease,” and “Gene Expression, Carbohydrate Metabolism, Small Molecule Biochemistry” (Table S4). The top five tox lists engaged “TGF-signaling,” “Liver Proliferation,” “Renal Necrosis/Cell Death,” “PARP/RXR Activation,” and “Mechanism of Gene Regulation by Peroxisome Proliferators via PPAR” (Table S5).

### Identification of *Mtb* Effectors Binding With Representative Human Protein Candidates

**Figure 5:**
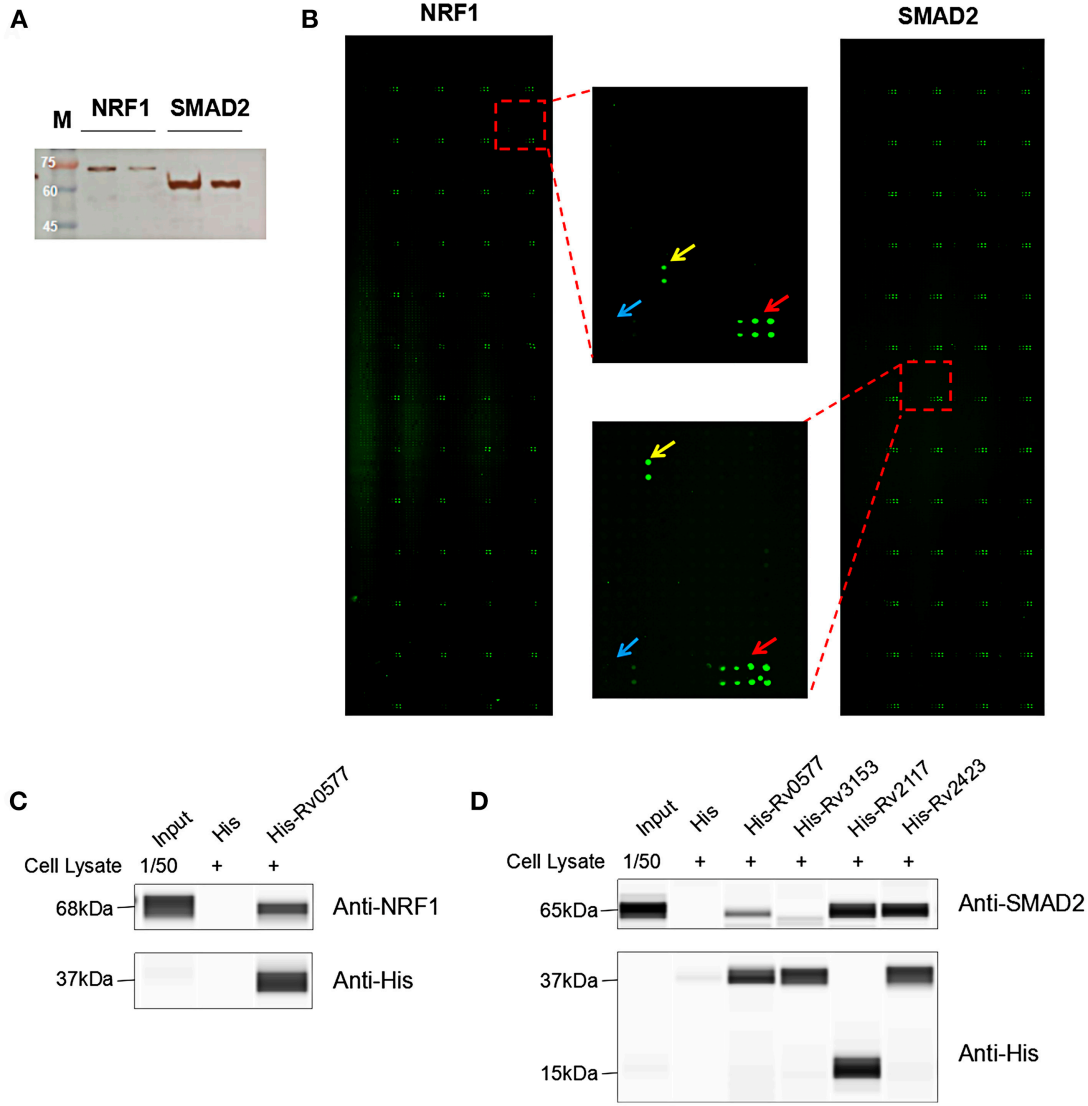
The NRF1 and SMAD2 interactors identified on a *Mtb* proteome microarray and validation by His pull-down assay. **(A)** Silver staining to detect the purified NRF1-myc and SMAD2-myc. **(B)** NRF1 and SMAD2 binding study with *Mtb* proteome microarray. Each array contained Cy3 labeled BSA as a positive control (marked with red arrows) and BSA as a negative control (marked with blue arrows). Positive proteins were marked with yellow arrows. The cutoff for calling positive candidates was set as signal-to-noise (SNR) ratio ≥ 1.4 and Z-score ≥ 3.0. **(C)** His tagged -Rv0577 pull-down assay with endogenously expressed NRF1. **(D)** His tagged -Rv0577, -Rv3153, -Rv2117, and -Rv2423 with endogenously expressed SMAD2, respectively. Protein separation and detection were performed using an automated capillary electrophoresis system. The results are representative of two independent experiments.

**Figure 6:**
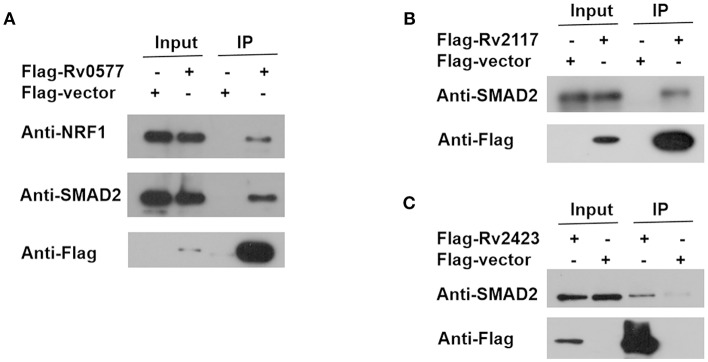
Co-IP of endogenous NRF1 with Rv0577 and endogenous SMAD2 with Rv0577, Rv2117, and Rv2423. **(A)** Co-IP of Flag-Rv0577 with endogenously expressed NRF1 and SMAD2. **(B,C)** Co-IP of Flag-Rv2117 and Flag-Rv2423 with endogenously expressed SMAD2, respectively. Western blot shown are representative of two independent experiments.

To identify the *Mtb* effectors interacting with host protein candidates, we selected two of the candidates NRF1 and SMAD2 as bait proteins (Supplementary Figure 2), which were both detected to be positive interactors with *Mtb* CP as well as SP with high SNR signals (SNR > 4). Meanwhile, SMAD2 was listed as one of the genes that were significantly enriched in the top 1 of IPA pathway item of “TGF-β Signaling” (Table S2), and NRF1 played an important role in the top 1 of IPA cellular function of “Activation of DNA endogenous promoter” (Table S3). As transcriptional factors, they play important roles in regulating many functional genes' expressions involved in various biological pathways, including stress adaptation and immune homeostasis (Sykiotis and Bohmann, 2010; Sekiya et al., 2016). First, myc-tagged NRF1 and SMAD2 were constructed, expressed, purified and detected by standard silver staining (Figure 5A). After Cy3 labeling, these two proteins were incubated with *Mtb* functional proteome microarray that was consisting of 3,829 and 433 proteins from Mtb strains H37Rv and CDC1551, respectively (Deng J. Y. et al., 2014). To identify potential candidates, the cut-off was set as the signal-to-noise ratio (SNR) > 1.4 and Z-score>3.0 (Figure 5B). We identified three potential NRF1 binding partners Rv2239c (Uncharacterized protein), Rv0577 (Putative glyoxylase Cfp32), Rv2499c (Possible oxidase regulatory-related protein) and six SMAD2 binding partners Rv0577, Rv2499c, Rv2423 (Methyltransferase type 12), Rv3241c (Ribosome hibernation promotion factor, Hpf), Rv3153 (NADH-quinone oxidoreductase subunit I, NuoI) and Rv2117 (Uncharacterized protein), of which Rv0577 and Rv2499c interacted with both NRF1 and SMAD2 (Table 2). Notably, Rv0577 had been identified as one of the *Mtb* secreted proteins and interacted with host TLR2 in a previous study (de Souza et al., 2011; Byun et al., 2012), while Rv2239c and Rv2423 were found to be *Mtb* effectors by He and his colleagues recently (He et al., 2017). In order to validate these interactions between *Mtb* effectors and NRF1 or SMAD2, we successfully purified His tagged Rv0577, Rv3153, Rv2117, and Rv2423 (Supplementary Figure 3), and incubated each of them with PMA-treated THP-1 lysate before performing His tagged pull-down. In this way, we found that endogenously expressed NRF1 could be enriched by His tagged Rv0577, meanwhile, SMAD2 could be enriched by His tagged Rv2117 and Rv2423, partially pulled down by Rv0577 but not binding to Rv3153 (Figures 5C,D, Supplementary Figures 1, 2). Further validation by Co-IP of Flag-Rv0577 with endogenouse NRF1 while Flag-Rv0577, -Rv2117 or -Rv2423 with endogenouse SMAD2 were also constant with the His pull-down results (Figure 6). Thus, Rv0577 was a key node for establishing a preliminary *Mtb*-human PPI network under the limited verification (Supplementary Figure 4). These results suggested that our strategy was effective to discover the *Mtb*-host protein-protein interactions which will facilitate to depict the complete interactome between the pathogen and host.

**Table 2:** The list of NRF1 and SMAD2 interactors in *Mtb* identified on a *Mtb* proteome microarray.

**Protein**	**Rv number**	**SNR**	**Z-score**	**Annotation**
NRF1	Rv2239c^*^	2	11.35	Uncharacterized protein
	Rv0577^#^	1.62	6.46	Putative glyoxylase Cfp32
	Rv2499c	1.4	3.54	Possible oxidase regulatory-related protein
SMAD2	Rv0577^#^	5.95	50.41	Putative glyoxylase Cfp32
	Rv2499c	1.6	5.24	Possible oxidase regulatory-related protein
	Rv2423^*^	1.51	4.36	Methyltransferase type 12
	Rv3241c	1.44	3.65	Ribosome hibernation promotion factor, Hpf
	Rv3153	1.42	3.38	NADH-quinone oxidoreductase subunit I, NuoI
	Rv2117	1.4	3.2	Uncharacterized protein

# Secreted proteins (de Souza et al., 2011).

* *Were identified as Mtb effectors (He et al., 2017)*.

## Discussion

The outcome of infection by *Mtb* largely depends on how the host responds to the invading bacteria and how the bacteria manipulates the host, which is facilitated by PPI. Elucidating protein interactions between human and *Mtb* would enable us to get insight into molecular mechanisms of pathogenesis. In the present study, we designed experiments to systematically determine all the human *Mtb* binding proteins and developed a strategy to identify *Mtb* effectors using an unbiased, comprehensive protein-chip based approach. Since extensive remodeling on the host is attributed to *Mtb* secreted virulence factors, cellular membrane proteins with access to the exterior, as well as part of cytosolic *Mtb* proteins released from lysate of bacterial cells inside phagocytes (He et al., 2017), we extracted *Mtb* SP and CP to cover most of *Mtb* effectors. According to human proteome microarray screening, a total of 84 interacting partners in a human host were successfully identified to bind with *Mtb* proteins. A bioinformatics analysis showed that these *Mtb* interactors were mainly related to gene expression, specifically transcription regulation. Further, they played roles in a wide range of cellular functions, such as activation of DNA endogenous promoters, transcription of DNA/RNA and necrosis, as well as signaling pathways, including TGF-β signaling and immune-related signaling pathways. What's more, to identify their *Mtb* effectors, we chose two protein candidates SMAD2 and NRF1 for further expression and incubation with *Mtb* proteome microarray, by which we screened for three binding partners for NRF1 and six interacting factors for SMAD2. Finally, His tagged pull-down assay and Co-IP verified several *Mtb* effectors, including Rv0577, Rv3153, Rv2117, and Rv2423.

To our knowledge, this is a pioneer experimental study on global human *Mtb* interactors, but has some similarities with a recent research (He et al., 2017) in which they globally profiled *Mtb* proteins interacting with proteins from macrophage by using *Mtb* proteome microarray. However, only membrane or secreted proteins were selected and considered as potential *Mtb* effectors. Besides, their human binders were identified by using GST-pull down followed by mass spectrometry analysis. Our strategy is differently based on a human proteome microarray containing 15,581 affinity-purified human proteins to profile potential *Mtb* interactors in human, and then reversely screening of their *Mtb* effectors by using *Mtb* proteome microarray. Specifically, there are several advantages of our two-way microarray strategy. First, it can globally and unbiasedly profile the bindings of more than 15,000 human proteins with *Mtb* SP and CP in a single experiment. Second, *Mtb* SP and CP, which comprise most *Mtb* effectors in a physiological condition, were both harvested and incubated with human proteome microarray to discover as much as *Mtb* interactors, not only membrane or secreted proteins. Third, all the proteins on the chips were overexpressed and purified under the same procedure, which means the local concentration of proteins on the microarray was relatively high, facilitating the PPI discovery. However, our strategy also has its limitation that we might have missed some positive interacting proteins not printed on microarray, and there are also lots of work related to validation that needs to be performed so as to get a comprehensive PPI network between *Mtb* and its human host.

To systematically explore the mechanism of *Mtb* pathogenesis in host cells, the functions and pathways that human *Mtb* interactors engaged in were analyzed. As expected, the presence of cancer related functions and pathways were detected in our analysis, as cancer shares many similarities with pathogen infection, such as evading immune response, tumor cell proliferation, death, metastasis, and invading cells. Among these overlapping pathways, couples of them have been experimentally tested and verified as having a role in tuberculosis. For example, TGF-β signaling pathway modulated T cell responses in early *Mtb* infection and facilitated inhibition of *Mtb* viability (Feruglio et al., 2017). Melatonin signaling was found to be effective in combating various bacterial and viral infections, including infections induced by *Mtb* (Srinivasan et al., 2012). IL-22 dependent pathways in both epithelial cells and macrophages mediate protective mechanisms for *Mtb* control (Treerat et al., 2017). Consistent with protein classification which showed these candidate proteins were classified into transcription factor, nucleic acid binding and receptor, our analysis results give a clue that *Mtb* manipulated immune and other related signaling pathways according to binding with receptors, transcription factors or DNA elements on the genome to regulate functional genes expression.

As expected, we found few overlaps between our newly identified *Mtb* interactors and currently known *Mtb* interactors (Table S1). While many reported interactors were discovered using different methods, such as Y2H or Mass-spec which have their own pros and cons, the results provided complementary types of information (Deng R. P. et al., 2014). Our discovery illustrated some novel potential molecules that were worthy of further exploring. For example, previous study has reported that activation and increased expression of PPARγ triggered by mycobacterial infection might aid the mycobacteria in circumventing the host response as an escape mechanism (Almeida et al., 2012). Recent published work indicated that PPARα was essential for antimycobacterial responses (Kim et al., 2017). PPARδ, as a sub-type of PPARs which mainly take part in the metabolism, cell proliferation and immune response, might also have important functions in response to *Mtb* infection. Indeed, it has been reported that PPARδ played a role in lipid absorption and metabolism (Luquet et al., 2005). MBD2, as a reader of DNA methylation, might contribute to interpret DNA methylation patterns into different functional output, including immune system function (Wood and Zhou, 2016). Although the role of MBD2 in tuberculosis is undefined, previous study reported that mycobacterial proteins could modify gene methylation in mammalian host tissues (Danjuma et al., 2017). For example, BCG-induced epigenetic reprogramming of immune cell function could enhance anti-mycobacterial immunity in macrophages (Verma et al., 2017). MAPK3 has been predicted to be an interactor in human in response to *Mtb* infection (Rapanoel et al., 2013). It is an enzyme which is a member of a MAPK family. Induction of the MAPK pathway is required for the expression of TNF-α, IL-10, and MCP-1 by human monocytes during *Mtb* infection (Song et al., 2003). SMAD2 is an intracellular signal transducer and transcriptional modulator. Formation of the SMAD2/SMAD4 complex, activates transcription. It has been reported that SMAD2 were up-regulated and absent in LTBI individuals (Stern et al., 2009). A recent authoritative study reported that NRF1 directly bound to and specifically sensed cholesterol in the endoplasmic reticulum (ER), acting as a central role in cholesterol homeostasis (Widenmaier et al., 2017). While previous study has indicated that *Mtb* could disturb cholesterol homeostasis in the host cell to support sustained infection (Vermeulen et al., 2017). Our results implied that *Mtb* might modulate this process by binding with NRF1. Combined with bioinformatics analysis results that most *Mtb* interactors identified in our study regulated gene expression, NRF1 and SMAD2 were selected to find their binding partners in *Mtb*. Finally, we found that Rv0577, which has been identified to be an interactor of TLR2 to induce maturation of dendritic cells and drives Th1 immune response (Byun et al., 2012), could bind with both NRF1 and SMAD2. Rv2423, having been identified as a *Mtb* effector (He et al., 2017), was also detected to be an interacting partner for SMAD2 in our study. Although His tagged Rv2239c were unsuccessfully purified at last, it has been detected as the top one potential binding protein with NRF1 in our microarray assay, which was also found to be *Mtb* effector by He et al. (2017).

Taken together, we have performed, to our knowledge, the first global protein chip analysis of human interactors with *Mtb* using the human proteome microarray and screened 84 proteins out of more than 15,000 proteins in human which might be responsible for pathogenesis in human after *Mtb* infection. In addition, we also verified the *Mtb* effectors of two representative proteins by reversely incubating with *Mtb* proteome microarray followed validation of His tagged pull-down assay and Co-IP, which will fill the gap in *Mtb*-human PPI data. However, there is far more verification work that needs to be done before we can show a comprehensive PPI network between *Mtb* and its human host. We believe that our results could serve as a valuable resource for both basic study and clinical research in the future.

## Data Availability

The raw data supporting the conclusions of this manuscript will be made available by the authors, without undue reservation, to any qualified researcher.

## Author Contributions

TC and ZZ conceived and designed the experiments. HJ, TC, and LL performed the experiments. LL analyzed the data and wrote the paper. JW, FD, LP, ZL, AX, YM, and JX provided suggestions on analysis and writing. ZZ wrote and revised the manuscript. All authors read and approved the final manuscript.

### Conflict of Interest Statement

The authors declare that the research was conducted in the absence of any commercial or financial relationships that could be construed as a potential conflict of interest.
