# Supplementary material for: A Two-Way Proteome Microarray Strategy to Identify Novel Mycobacterium tuberculosis-Human Interactors
Source: Front Cell Infect Microbiol. 2019 Mar 28;9:65. doi: 10.3389/fcimb.2019.00065 (PMC6448480; doi:10.3389/fcimb.2019.00065)
Supplement: Supplementary file 1 [file Data_Sheet_1.pdf]

Supplementary Material

A Two-way Proteome Microarray Strategy to Identify Novel *Mycobacterium tuberculosis*-human Interactors

Tingming Cao[1#], Lingna Lyu[1#], Hongyan Jia[1], Jinghui Wang[1], Fengjiao Du[1], Liping Pan[1], Zihui Li[1], Aiying Xing[1], Jing Xiao[1], Yu Ma[1], Zongde Zhang[1]*

1 Beijing Key Laboratory for Drug Resistant Tuberculosis Research, Beijing Tuberculosis and Thoracic Tumor Research Institute, Beijing Chest Hospital, Capital Medical University, Beijing, China.

[#] These authors contributed equally to this work.

* **Correspondence:** Corresponding Author: zzd417@ccmu.edu.com

1 Supplementary Figures and Tables

1.1 Supplementary Figures

A

Protein	SNR534
IKZF4	11.24
SOX10	10.97
IRF2	5.44
BARX2	4.47
ZFP91	3.64
GRM8	3.13
MBD2	2.98
PPARD	2.50
FOXD3	2.34
ZNF70	2.18

B

Protein	SNR647
C6orf166	7.62
LDHA	7.11
LAPTM4A	6.69
CYP39A1	6.39
CSRP2BP	6.22
PARVA	6.14
ITM2B	5.92
CPA1	5.21
SF3B2	4.92
NDUFB6	4.92
BCL2L14	4.86
MBTPS1	4.77
GNB1L	4.57
ACP1	3.78
PPCDC	3.65
MAPK3	3.62
FLJ20366	3.44
RFC4	3.27
GLO1	3.23
CD300LB	3.16
VPS8	3.12
SGCA	3.04
PCK2	2.79
BAT1	2.55
CDX2	2.49
NFE2L2	2.43
SLC39A8	2.40
HNF4A	2.31
RORA	2.21
SOX4	2.20

C

Protein	SNR534	SNR647
MAP2K5	16.05	54.80
HOXB4	9.59	13.92
MTA2	8.33	3.66
ZNF253	7.47	6.78
NRF1	6.53	8.98
OVOL1	6.32	7.02
INSM1	6.21	7.59
ZNF700	6.01	7.86
ZNF540	5.74	10.38
HOXB9	5.69	3.75
HOXB5	4.97	3.67
KIAA2018	4.96	5.68
ZNF343	4.63	8.33
SRCAP	4.33	12.72
GMEB2	4.27	4.22
SMAD2	4.24	5.90
ZNF23	4.23	3.72
ZNF100	4.17	3.49
ZNF425	3.98	4.02
ZNF557	3.97	4.16
GLIS2	3.89	13.95
SRY	3.72	2.06
ZNF570	3.63	3.73
ZBTB12	3.56	2.27
FOXM1	3.52	2.19
ZNF541	3.51	5.07
DPRX	3.48	2.82
VENTX	3.47	3.45
RNF113B	3.40	4.53
SP6	3.37	6.68
ZNF337	3.33	3.31
SMAD4	3.10	4.53
ZNF699	2.92	3.51
ZNF136	2.76	2.34
TSC22D4	2.67	2.74
IKZF3	2.62	3.00
ESRRG	2.59	2.57
ZNF569	2.54	3.52
ZNF410	2.41	3.43
KLF8	2.39	2.62
ARID3A	2.31	2.54
KLF11	2.27	2.90
STAT5B	2.06	2.73
SKOR2	2.04	3.44

Supplementary Figure 1. *Mtb* SP-unique, CP-unique and SP-CP-common interactors screened on human proteome microarray. (A) *Mtb* SP-unique interactors (green), **(B)** *Mtb* CP-unique interactors (red) and **(C)** *Mtb* SP-CP-common interactors were showed with their SNR≥2.0. The colors shades represent the numerical value.

Protein	Image	SNR532	Image	SNR647
NRF1	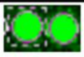	6.53	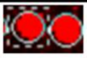	8.98
SMAD2	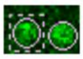	4.24	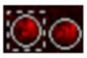	5.90

Supplementary Figure 2. NRF1 and SMAD2 are potential *Mtb* interactors. Potential *Mtb* interactors NRF1 and SMAD2 were set as representative proteins for further study. The SNR and images were shown. Experiments were performed in two replicates.

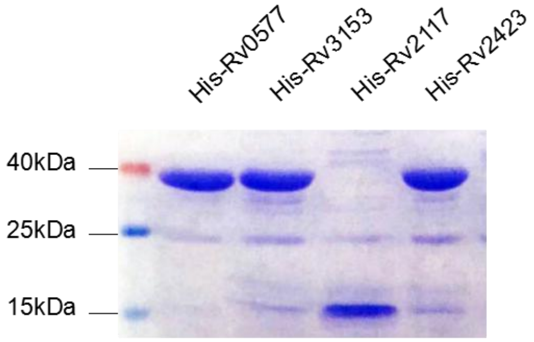

Supplementary Figure 3. Purification of His tagged Rv0577, Rv3153, Rv2117 and Rv2423. His tagged *Mtb* proteins were purified and detected by SDS-PAGE followed by coomassie brilliant blue staining.

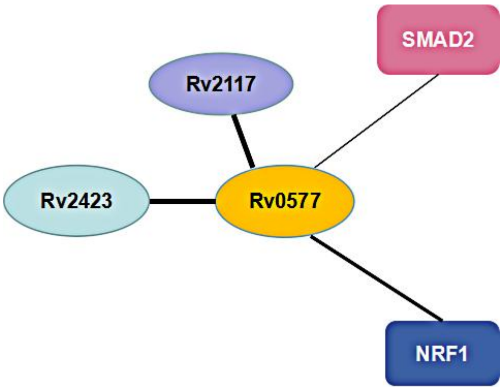

Supplementary Figure 4. PPI based on the experimental validation. Network plot was visualized by Cytoscape software. Nodes depict proteins (rectangle: human proteins, ellipse: *Mtb* proteins) by various colors and edges represent interactions.

1.2 Supplementary Tables

Table S1 The host interacting proteins with *Mtb* that have been identified from previous studies.

Host Protein Name	Mtb binding partners	Uniprot ID	Method	Reference
TRIM27	PtpA	P14373	Y2H	Wang J. et al., 2016, *Sci Rep*
IL8	Rv3248c,Rv0296c,Rv1018c	P10145	AP/MS	Wang J.et al., 2015, *Nat Immunol*
Ub	PtpA		Y2H	Dziadek B.et al., 2016, *Plos One*
β2M	ESAT-6	P61769	Y2H	Sreejit G. et al., 2014, *Plos Pathog*
Hrs	EsxH,EsxG	O14964	Y2H	Mehra A.et al., 2013, *Plos Pathog*
DUSP16 /MKP-7	Eis	Q9BY84	acetylation assays *in vitro*	Kim K.H.et al., 2012, *PNAS*
TLR2	Rv0577	O60603	Pulldown	Byun E.H.et al., 2012, *FASEB J*
V-ATPase	PtpA	Q15904	Pulldown	Wong D.et al., 2011, *PNAS*
Rab5/Rab7	NdkA	P20339/P51149	Pulldown	Sun J.et al., 2010, *Plos One*
ALO17	Rv3655c	Q63HN8	Pulldown	Danelishvili L.et al., 2010, *Plos One*
PSF	Rv3654c	P23246	Pulldown	Danelishvili L.et al., 2010, *Plos One*
VPS33B	PtpA	Q9H267	substrate trapping	Bach H.et al., 2008, *Cell Host Microbe*
Coronin-1A	LpdC	P31146	Pulldown and 2D-gel	Deghmane A-E.et al., 2007, *J Cell Sci*
PI3P	SapM	Q13496	phosphatase assay *in vitro*	Vergne I.et al., 2005, *PNAS*

Complement C3	HbhA	P01024	AP/MS	Mueller-Ortiz S.L. et al., 2001, *Infect Immun*

Table S2 Top twenty IPA pathways for *Mtb* interactors

Ingenuity Canonical Pathways	-log(p-value)	Ratio	Molecules
TGF-β Signaling	3.47	0.046	SMAD2,MAPK3,SMAD4,HNF4A
Transcriptional Regulatory Network in Embryonic Stem Cells	2.94	0.0556	CDX2,HNF4A,FOXD3
Role of NANOG in Mammalian Embryonic Stem Cell Pluripotency	2.92	0.0328	MAPK3,CDX2,SMAD4,FOXD3
Osteoarthritis Pathway	2.89	0.0236	SMAD2,GLIS2,PPARD,SMAD4,SLC39A8
Melatonin Signaling	2.59	0.0417	RORA,MAPK3,MAP2K5
Ephrin B Signaling	2.57	0.0411	MAPK3,ACP1,GNB1L
Regulation of IL-2 Expression in Activated and Anergic T Lymphocytes	2.46	0.0375	SMAD2,MAPK3,SMAD4
IL-22 Signaling	2.43	0.0833	MAPK3,STAT5B
Role of JAK family kinases in IL-6-type Cytokine Signaling	2.39	0.08	MAPK3,STAT5B
Antiproliferative Role of TOB in T Cell Signaling	2.36	0.0769	SMAD2,SMAD4
PPARα/RXRα Activation	2.31	0.0222	SMAD2,MAPK3,SMAD4,STAT5B
Sirtuin Signaling Pathway	2.29	0.0171	PCK2,MAPK3,NDUFB6,LDHA,NFE2L2

Acute Myeloid Leukemia Signaling	2.27	0.0323	MAPK3,STAT5B,MAP2K5
PPAR Signaling	2.25	0.0316	PPARD,MAPK3,STAT5B
Regulation of the Epithelial-Mesenchymal Transition Pathway	2.23	0.0212	SMAD2,MAPK3,SMAD4,MAP2K5
Oncostatin M Signaling	2.13	0.0588	MAPK3,STAT5B
DNA Methylation and Transcriptional Repression Signaling	2.13	0.0588	MTA2,MBD2
Chronic Myeloid Leukemia Signaling	2.12	0.0283	MAPK3,SMAD4,STAT5B
Mouse Embryonic Stem Cell Pluripotency	2.12	0.0283	MAPK3,SMAD4,FOXD3
Glucocorticoid Receptor Signaling	2.03	0.0147	SMAD2,PCK2,MAPK3,SMAD4,STAT5B

Table S3 Top ten IPA "Diseases and Bio Functions" items for *Mtb* effectors

Categories	Diseases or Functions Annotation	p-Value	Molecules	# Molecules
Gene Expression	Activation of DNA endogenous promoter	2.54E-20	AKIRIN2,ARID3A,BARX2,CDX2,ESRRG,FOXD3,FOXM1,GLIS2,GLO1,GMEB2,HNF4A,HOXB4,HOXB5,HOXB9,IKZF3,IKZF4,INSM1,IRF2,KLF11,KLF8,MAP2K5,MAPK3,MBD2,MTA2,NFE2L2,NRF1,OVOL1,PPARD,RORA,SKOR2,SMAD2,SMAD4,SOX10,SOX4,SP6,SRCAP,STAT5B,VENTX,ZNF136	39
Gene Expression	Transcription of DNA	1.61E-19	AKIRIN2,ARID3A,BARX2,CDX2,ESRRG,FOXD3,FOXM1,GLIS2,GLO1,GMEB2,HNF4A,HOXB4,HOXB5,HOXB9,IKZF3,IKZF4,INSM1,IRF2,KLF11,KLF8,MAP2K5,MAPK3,MBD2,	42

			MTA2,NFE2L2,NRF1,OVOL1,PPARD,RORA,SKOR2,SMAD2,SMAD4,SOX10,SOX4,SP6,SRCAP,SRY,STAT5B,VENTX,ZNF136,ZNF425,ZNF540	
Gene Expression	Transcription of RNA	1.06E-16	AKIRIN2,ARID3A,BARX2,CDX2,ESRRG,FOXD3,FOXM1,GLIS2,GLO1,GMEB2,HNF4A,HOXB4,HOXB5,HOXB9,IKZF3,IKZF4,INSM1,IRF2,KLF11,KLF8,MAP2K5,MAPK3,MBD2,MTA2,NFE2L2,NRF1,OVOL1,PPARD,RORA,SKOR2,SMAD2,SMAD4,SOX10,SOX4,SP6,SRCAP,SRY,STAT5B,VENTX,ZNF136,ZNF425,ZNF540	42
Cell Death and Survival	Necrosis	0.000000361	ACP1,AKIRIN2,BCL2L14,CD300LB,CDX2,FOXD3,FOXM1,GLIS2,GLO1,HNF4A,HOXB4,HOXB9,IKZF3,INSM1,IRF2,ITM2B,KLF8,LDHA,MAP2K5,MAPK3,MBTPS1,MTA2,NFE2L2,NRF1,PARVA,PCK2,PPARD,SF3B2,SGCA,SMAD2,SMAD4,SOX10,SOX4,SRCAP,STAT5B	35
Gene Expression	Transactivation of RNA	0.000000606	CD300LB,ESRRG,GLIS2,HNF4A,IRF2,MAPK3,NFE2L2,PARVA,PPARD,RORA,SMAD2,SMAD4,SOX10,STAT5B	14
Cellular Movement	Invasion of breast cancer cell lines	0.00000151	BARX2,FOXM1,KLF8,LDHA,MBD2,PARVA,RORA,SMAD2,SMAD4,SOX4	10
Cellular Movement	Invasion of cells	0.00000197	BARX2,CDX2,FOXD3,FOXM1,HOXB9,KLF8,LDHA,MAP2K5,MBD2,NFE2L2,PARVA,PPARD,RFC4,RORA,SMAD2,SMAD4,SOX4,STAT5B	18
Embryonic Development, Organismal Development	Patterning of rostrocaudal axis	0.00000673	CDX2,HOXB4,HOXB5,HOXB9,SMAD2,SMAD4	6
Cellular Development, Cellular Growth and Proliferatio	Cell proliferation of tumor cell lines	0.00000774	AKIRIN2,ARID3A,BARX2,CDX2,ESRRG,FOXD3,FOXM1,HNF4A,HOXB9,IRF2,LDHA,MAPK3,MBD2,MTA2,NFE2L2,PCK2,PPARD,RFC4,RORA,SMAD2,SMAD4,SOX4,STAT5B,V	24

n			ENTX	
Cell Death and Survival	Cell death of tumor cell lines	0.0000107	BCL2L14,CDX2,FOXD3,FOXM1,GLO1,HNF4A,HOXB9,IRF2,KLF8,MAP2K5,MAPK3,MTA2,NFE2L2,NRF1,PARVA,PCK2,PPARD,SMAD2,SMAD4,SOX10,SOX4,SRCAP,STAT5B	23

Table S4 Top three networks in diseases and functions for *Mtb* interactors

Top network functions	Score	Focus Molecules
Gene Expression, Embryonic Development, Organismal Development	32	AKIRIN2,CDX2,BCL2L14,CYP39A1,FOXD3,HOXB4,HOXB9,MAP2K5,NFE2L2,PCK2,RORA,SF3B2,SKOR2,SMAD2,SMAD4,SRCAP
Cellular Assembly and Organization, Endocrine System Disorders, Gastrointestinal Disease	32	ACP1,DDX39B,GLO1,GNB1L,HNF4A,KLF11,LAPTM4A,NDUFB6,NRF1,OVOL1,PARVA,SOX4,SOX10,STAT5B,ZNF410,ZNF557
Gene Expression, Carbohydrate Metabolism, Small Molecule Biochemistry	27	ARID3A,ESRRG,FOXM1,IKZF3,IKZF4,INSM1,IRF2,KAT14,LDHA,MAPK3,MBD2,MTA2,PPARD,VPS8

Table S5 Top five tox functions list for *Mtb* interactors identified

Name	p-value	overlap
TGF- Signaling	3.84E-04	4.4 % 4/90
Liver Proliferation	2.13E-03	2.1 % 5/238
Renal Necrosis/Cell Death	4.84E-03	1.3 % 7/549
PPAR/RXR Activation	5.20E-03	2.2 % 4/183
Mechanism of Gene Regulation by Peroxisome Proliferators via PPAR	5.67E-03	3.2 % 3/95

1.3 Supplementary Original Figures

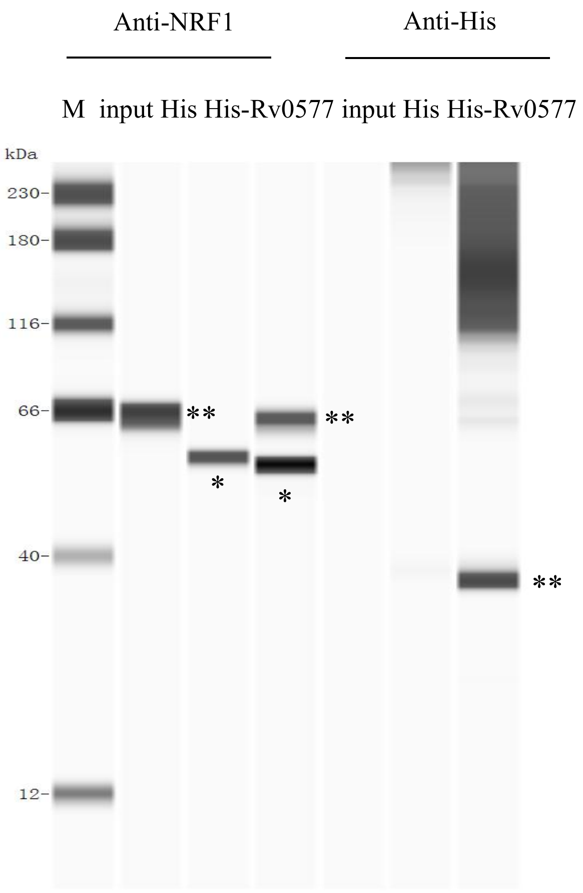

Supplementary Original Figure 1. **His pull-down of His-Rv0577 with endogenous NRF1.** ** represented specific band, * represented non-specific band

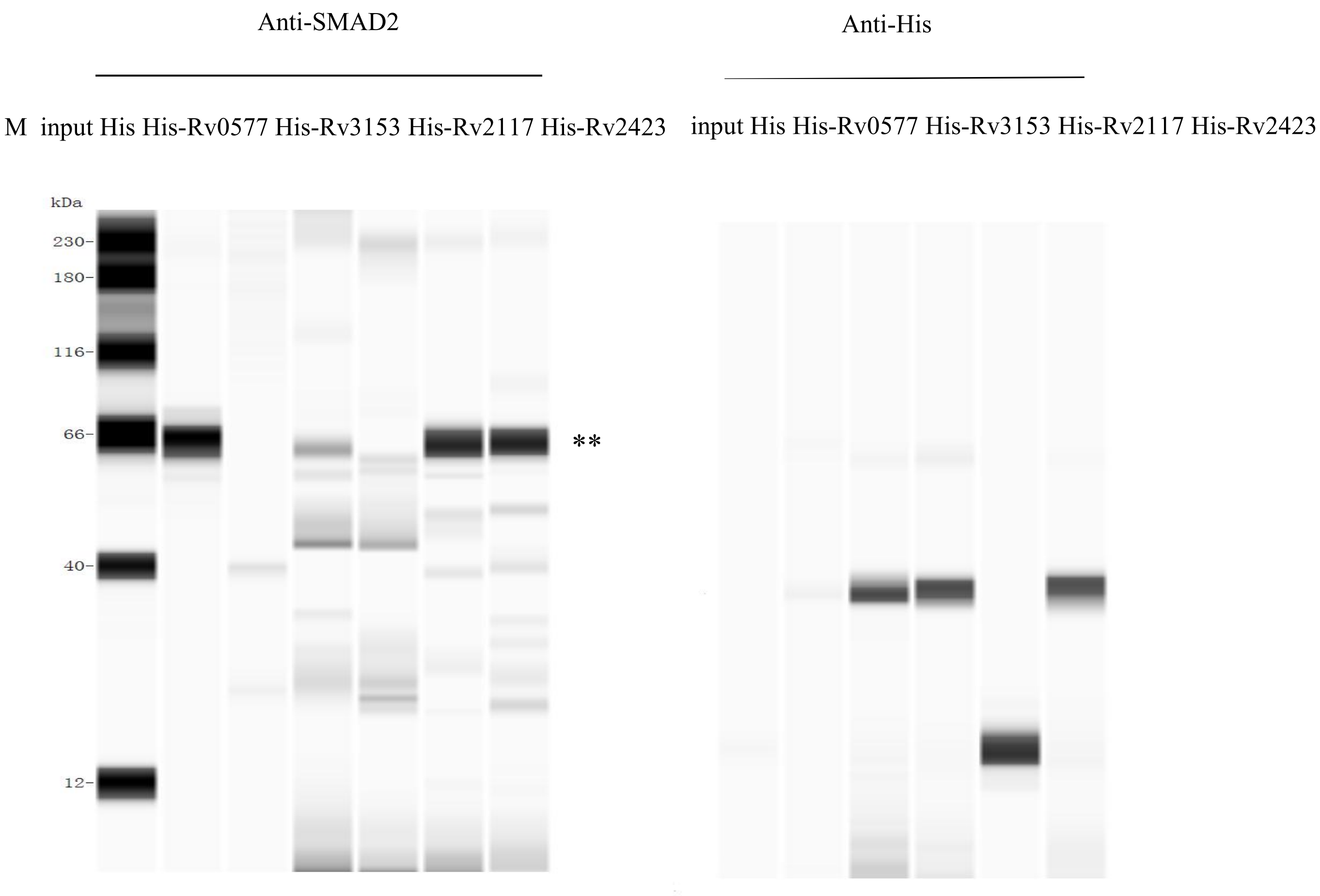

Supplementary Original Figure 2. **His pull-down of His-Rv0577, His-Rv3153, His-Rv2117 and His-Rv2423 with endogenous SMAD2.** ** represented specific band

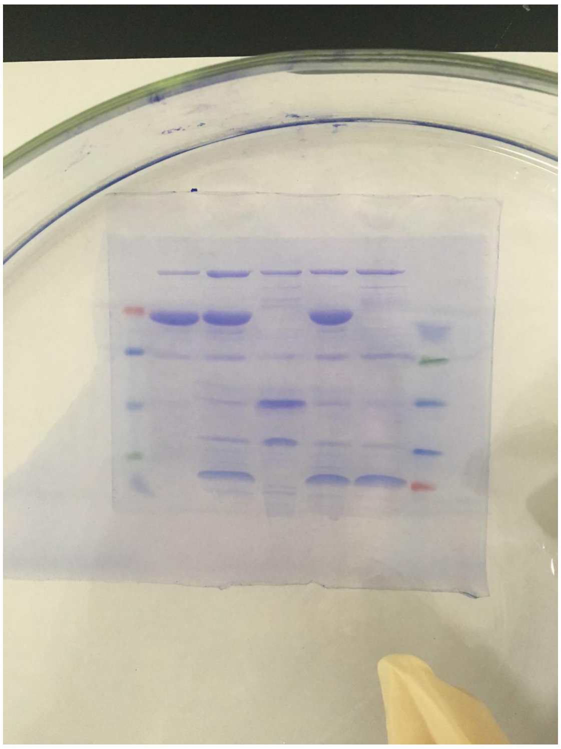

Supplementary Original Figure 3. Purification of His tagged Rv0577, Rv3153, Rv2117 and Rv2423.

frontiers
